# The role of oral microbiota in cancer

**DOI:** 10.3389/fmicb.2023.1253025

**Published:** 2023-10-24

**Authors:** Zhou Lan, Wei-Jia Liu, Hao Cui, Ke-Long Zou, Hao Chen, Yu-Yue Zhao, Guang-Tao Yu

**Affiliations:** ^1^Stomatological Hospital, School of Stomatology, Southern Medical University, Guangzhou, China; ^2^Department of Oral Mucosal Diseases, Guangzhou Key Laboratory of Basic and Applied Research of Oral Regenerative Medicine, Affiliated Stomatology Hospital of Guangzhou Medical University, Guangzhou, China

**Keywords:** oral microbiota, cancer, tumor, inflammation, tumor microenvironment

## Abstract

Cancer remains a significant global challenge, with an estimated 47% increase in cancer patients from 2020 to 2040. Increasing research has identified microorganism as a risk factor for cancer development. The oral cavity, second only to the colon, harbors more than 700 bacterial species and serves as a crucial microbial habitat. Although numerous epidemiological studies have reported associations between oral microorganisms and major systemic tumors, the relationship between oral microorganisms and cancers remains largely unclear. Current research primarily focuses on respiratory and digestive system tumors due to their anatomical proximity to the oral cavity. The relevant mechanism research mainly involves 47% dominant oral microbial population that can be cultured *in vitro*. However, further exploration is necessary to elucidate the mechanisms underlying the association between oral microbiota and tumors. This review systematically summarizes the reported correlations between oral microbiota and common cancers while also outlining potential mechanisms that may guide biological tumor treatment.

## 1. Introduction

Cancer is the leading cause of mortality worldwide, with an estimated global population of 24.8 million cancer patients projected for 2040 (Sung et al., 2021). Female breast cancer has emerged as the most prevalent malignancy globally, followed by lung, liver, stomach and colorectal cancer (CRC) (Cao et al., 2021). During tumor malignant transformation, certain characteristics that contribute to tumor progression may be acquired, such as indefinite replicative potential and incorporation of polymorphic microbiomes (Hanahan, 2022). The understanding of tumors is expanding alongside advancements in Shotgun metagenomic sequencing technique which have facilitated investigations into diverse microbial communities within tumors (Meslier et al., 2022). Over 3 × 10^3^ species of microorganisms are found on the mucosal surface, with more than 90% residing in the colon (Xiao et al., 2020; Sepich-Poore et al., 2021). Currently, only 11 species have been identified as carcinogenic microorganisms: seven viruses, three parasites and a single bacterium known as *Helicobacter pylori* (*H. pylori*) (Cullin et al., 2021).

**Figure 1:**
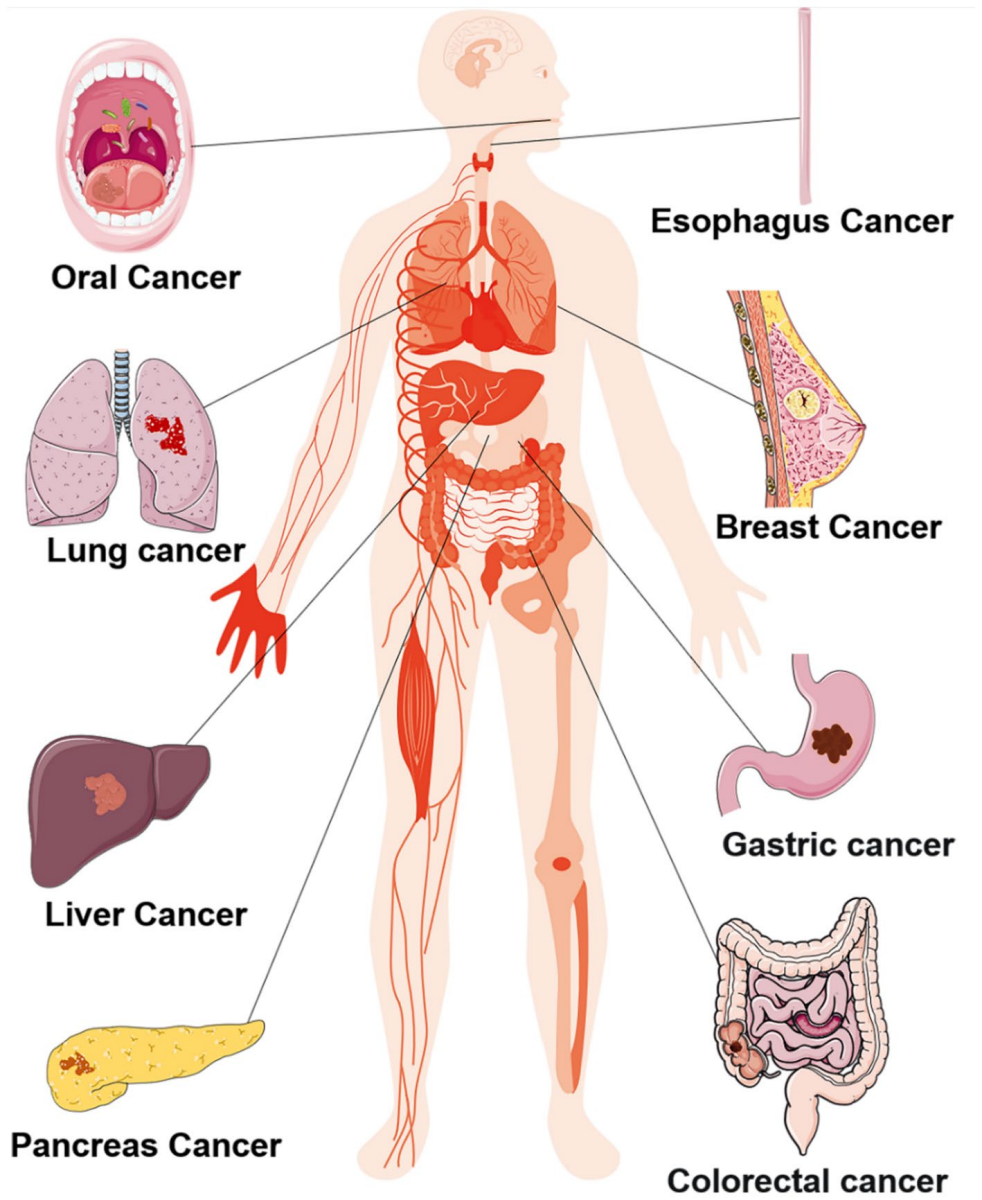
Oral microbiome associated major systemic tumors.

The oral cavity maintains an optimal temperature of 37°C and a pH range of 6.5–7.5, creating an exceptionally favorable environment for the survival of oral microorganisms (Koliarakis et al., 2019). According to the human oral microbiome database, more than 700 bacterial species inhabiting various ecological niches within the oral cavity (Gao et al., 2018; Koliarakis et al., 2019; Mark Welch et al., 2020). These oral microorganisms mutually benefit each other and collectively maintain the homeostasis of the oral ecosystem, however, this delicate balance is disrupted under pathological conditions (Li X. et al., 2022). Dysbiosis typically manifests as alterations in microbial composition characterized by a decrease in beneficial bacteria and an increase in potentially pathogenic bacteria (DeGruttola et al., 2016). Furthermore, dysbiosis not only give rise to local lesions such as caries and periodontal disease but also exerts systemic effects on distant organs, contributing to systemic diseases including cancers shown as Figure 1 (Sedghi et al., 2021). The main mechanism facilitating translocation of oral microorganisms include: (i) Interconnected anatomical structure between the oral cavity, respiratory tract and digestive tract allow for entry of oral microorganisms into these systems through saliva, air inhalation and food ingestion; (ii) Hematogenous and lymphogenous transmission: traumatic events like tooth extraction can result in invasion of blood circulation by oral microorganisms leading to distant metastasis (Mo et al., 2022).

**Figure 2:**
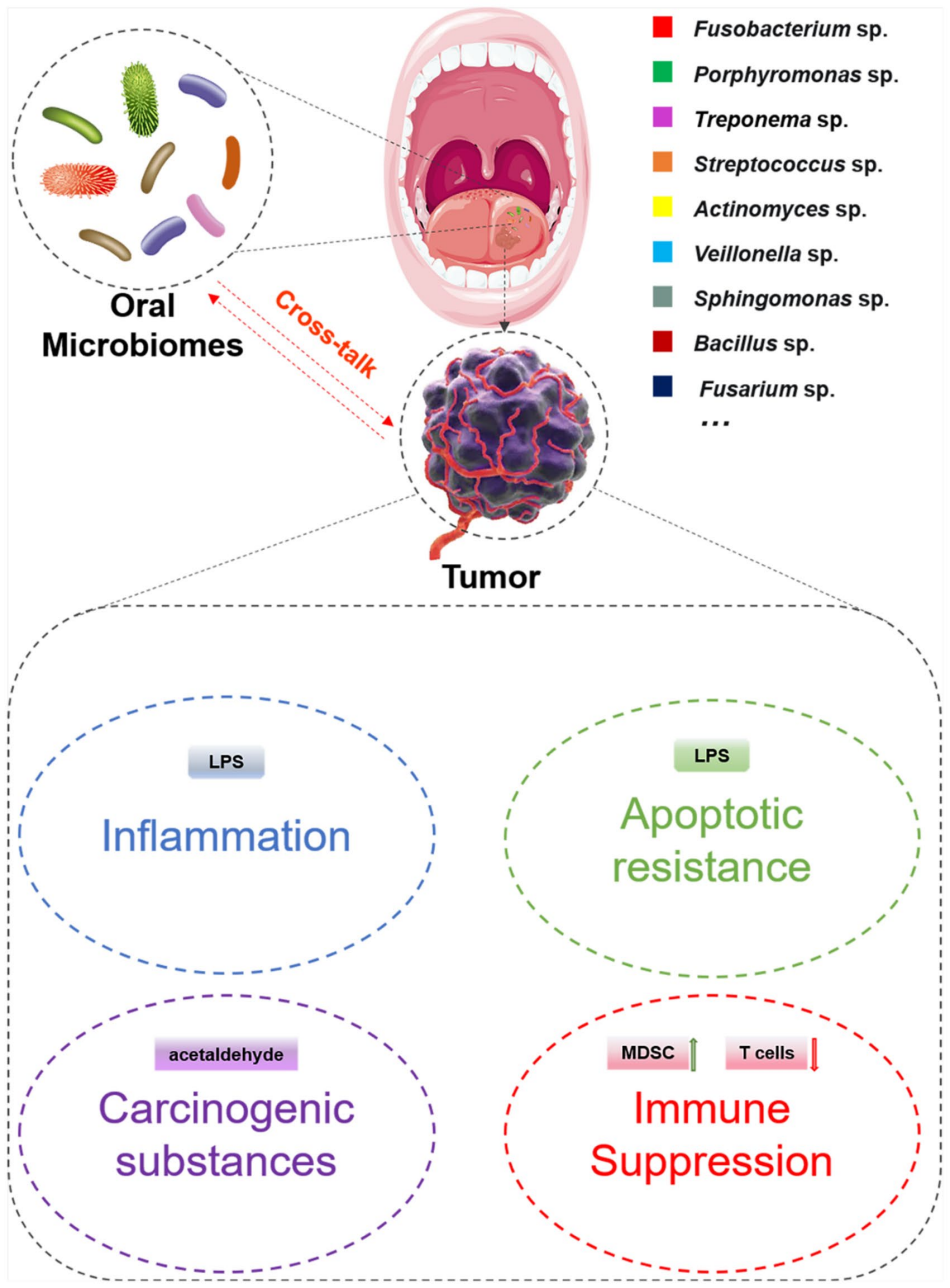
The potential mechanism associated with the cross-talk between oral microbiomes and tumors.

According to the existing researches, the mechanisms by which oral microbes promote tumor occurrence and development are as follows: (i) Induce and aggravate chronic inflammation and promote the occurrence and development of tumors by the components of themselves (Gagliani et al., 2014; Niklander, 2021). (ii) Regulate cells proliferation and apoptosis by disrupting cell cycle and tumor signal transduction (Meurman, 2010; Deo and Deshmukh, 2020; Bakhti and Latifi-Navid, 2021). (iii) Indirectly metabolize substances, including sulfides, nitrosamines, hydroxyl radical, acetaldehyde, deoxycholic acid and toxins, which interfere with tumor occurrence, metastasis and recurrence (Doherty and Cleveland, 2013; Belcheva et al., 2014; Teles et al., 2020). (iv) Regulate host immune response (Figure 2). Common oral microorganisms, such as *Fusobacterium nucleatum* (*F. nucleatum*) and *Porphyromonas gingivalis* (*P. gingivalis*), can promote the infiltration of immunosuppressive cells and interfere with the function of immune killer cells, thereby protecting tumor cells from immune system surveillance and clearance (Steffen et al., 2000; Colucci, 2015; Wen et al., 2020). In summary, the objective of this review is to provide a comprehensive analysis of the interface between oral microbiota and systemic cancer, while elucidating the underlying mechanisms involved.

## 2. Oral microbiome and cancer

### 2.1. Lung carcinoma

Lung carcinoma has the highest mortality rate at around 18%, equivalent to 1,796,144 cases (Sung et al., 2021). Smoking is the primary etiological factor for lung cancer, while other contributing factors include air pollution and infection (Liu and Dong, 2021). Numerous studies have provided evidence supporting a close association between lung cancer and carcinogenic viruses such as HPV and HIV, which is significantly elevate the risk of developing lung cancer (Zhai et al., 2015; Cribbs et al., 2020). Although HPV and HIV can also be detected in the oral cavity, they do not constitute the dominant microbiota. Prevailing bacteria species in the oral cavity encompass *P. gingivalis*, *F. nucleatum*, *Treponema dentinosum* and *Streptococcus* (Zhang et al., 2019). However, the relationship between lung cancer and major oral microorganisms has mostly been constrained to observational studies. Shi et al. (2021) discovered a strong correlation between poor oral hygiene resulting from periodontal disease and an increased susceptibility of lung cancer. Utilizing sequencing technology in cohort studies has revealed higher alpha diversity within oral microbes is associated with a reduced risk of developing lung cancer, and they also identified distinct representative microbial genera as potential indicators for monitoring lung cancer (Hosgood et al., 2021; Vogtmann et al., 2022; Zhou et al., 2022). It is evident that oral microorganisms and their microbial derivatives, including proteins, endotoxins, and other metabolites, can be transmitted to the respiratory tract or even through direct inhalation or hematogenous dissemination, thereby influencing the occurrence and progression of lung cancer (Ma et al., 2023). Furthermore, certain oral microbiota can directly induce chronic inflammation, immune and activation of carcinogenic signaling pathways in order to promote the development of lung cancer. Tsay conducted an analysis of airway rinse samples of 39 lung cancer patients and 36 non-cancer patients, revealing a higher abundance of *Streptococcus* and *Veillonella* in the lower airways of lung cancer patients. Furthermore, through transcriptome analysis of airway epithelial cells and *in vitro* experiments, they revealed that *Streptococcus* and *Veillonella* were related to the up-regulation of ERK and PI3K signaling pathways during carcinogenesis (Tsay et al., 2018). Similarly, Yang et al. (2018) identified an enrichment of *Sphingomonas* and *Bacillus* in the saliva of non-smoking female lung cancer patients, suggested that dysbiosis in oral saliva microbiota may regulate the apoptosis of lung cancer cells through the p53 pathway. But the limitation of these studies is the absence of *in vivo* experiments. It is noteworthy that *Helicobacter pylori* (*H. pylori*), the bacterium responsible for stomach cancer, can also be detected in the oral cavity (Zhang et al., 2022). Oral *H. pylori* has the potential to induce significant and persistent inflammation in the lung lining through direct inhalation or hematogenous transfer to bronchus or lung tissue, thereby promoting malignant transformation and tumor growth (GonzÁlez et al., 2018). The presence of *H. pylori* infection can potentially impact the efficacy of immunotherapy in patients with non-small cell lung cancer, and mechanistic investigations have elucidated that *H. pylori* has the ability to modulate dendritic cell cross-presentation activity, suppress CD8^+^ T cell response against tumors, and influence both innate and adaptive immune responses in the host (Oster et al., 2022). It is undeniable that the mechanism of oral microbiota and lung cancer is still in its infancy, and more exploration is needed in the future.

### 2.2. Colorectal cancer

CRC ranks the second in the world with a mortality rate of 9.4% (Sung et al., 2021). Colorectal mucosa harbors a diverse microbial community, and emerging evidence suggests a close association between CRC and gastrointestinal (GI) microbes (Tilg et al., 2018). However, the colonization of oral microbiota in the gut is hindered by the presence of an oral-gut barrier (Tugizov, 2016; Moutsopoulos and Konkel, 2020). Successful translocation of oral microorganisms to the gut requires overcoming two major challenges: (i) Traversing the upper digestive tract chemical barrier composed of acid and bile and (ii) surpassing intestinal colonization resistance mediated by commensal bacteria (Buffie and Pamer, 2013; Mo et al., 2022). Once this barrier is breached, ectopic colonization of oral microorganisms in the colorectal site can disrupt microbial ecology and lead to the occurrence of colorectal inflammation, thus creating an environment conducive to tumor progression (Reitano et al., 2021). The latest mechanistic investigations have revealed that oral microorganisms exert additional detrimental effects on the colon through their hydrolysis of glycoproteins present on the surface of colonic epithelial cells, degradation of mucin and extracellular matrix components, as well as synthesis of carcinogenic metabolites, reactive oxygen species, and polyamines (Cueva et al., 2020). Currently, numerous literatures have reported that intestinal dysbiosis is directly or indirectly related to the CRC (Gao et al., 2015; Wirbel et al., 2019). Moreover, oral and pharyngeal swabs, saliva, fecal samples and tumor tissue samples were used to reveal significant differences in oral microbial composition between CRC patients and healthy controls, which provides biological markers for the diagnosis of CRC (Kostic et al., 2013; Gao et al., 2015; Guven et al., 2019; Zhang et al., 2020; Wang Y. et al., 2021; Yu et al., 2022). Although the utility of throat swabs in assessing microbial composition has been subject to scrutiny, their sensitivity significantly increased from 53 to 76% with the inclusion of fecal samples (Flemer et al., 2018), but its universality needs to be further studied.

Cumulative evidences demonstrated that oral microorganisms, including but are not limited to *P. gingivalis*, *F. nucleatum* and *Streptococcus*, exhibit significantly increased abundance in tumors and feces of patients with CRC (Ahn et al., 2013; Kostic et al., 2013; McCoy et al., 2013; Drewes et al., 2017; Uchino et al., 2021). Two studies have similarly found that *P. gingivalis* can not only mediate the occurrence of enteritis by activating the NLRP3 inflammasome, but also activate the MAPK/ERK signaling pathway (Mu et al., 2020; Wang X. et al., 2021). In addition, it has been reported that tissue-infiltrating *P. gingivalis* can colonize dendritic cells and macrophages to escape clearance of immune system, resulting in systemic dissemination (Carrion et al., 2012). Approximately 40% infiltrated *F. nucleatum* in CRC originates from the oral cavity (Komiya et al., 2019). *F. nucleatum* has emerged as a major driver of CRC due to its anaerobic nature, high invasiveness, FAP2-dependent colorectal adhesion, and glucose free metabolism (Drewes et al., 2017; Osman et al., 2021). Moreover, its high abundance within CRC is associated with tumor metastasis, recurrence, chemotherapy resistance and reduced efficacy of radiotherapy (Yu et al., 2017; Dong et al., 2021; Chen S. et al., 2022; Ou et al., 2022). The potential mechanisms underlying these effects include: (i) Directly influencing tumor cells by regulating tumor metabolism and enhancing the stemness of cancer stem cells; (ii) Modulating T cell-mediated immunity and recruiting myeloid-derived suppressor cells to mediate tumor immune suppression; (iii) Producing inflammatory factors that create a pro-inflammatory microenvironment to promote CRC progression; (iv)Targeting TLR4/MyD88 signaling pathway to induce autophagy to promote chemotherapy resistance of CRC (Kostic et al., 2013; Yu et al., 2017; Ou et al., 2022). Interestingly, *F. nucleatum* has been found to activate the STING signaling pathway and up-regulate the expression of PD-L1, thus exhibiting a remarkable sensitivity toward PD-L1 immune blockade therapy (Gao Y. et al., 2021). Additionally, *Streptococcus* has been implicated in mediating inflammation and induce an immunosuppressive tumor microenvironment (TME) dominated by myeloid-derived immunosuppressive cells (MDSC) and tumor-associated macrophages, thereby promoting CRC progression (Long et al., 2019; Taylor et al., 2021).

### 2.3. Hepatocellular cancer

Hepatocellular cancer (HCC) ranks third in terms of mortality rate, with 830,180deaths (4.7%) reported (Sung et al., 2021). Well-established risk factors for high incidence of HCC include alcohol consumption and infection (Liu and Dong, 2021). However, limited knowledge exists regarding the association between oral microbiota and HCC. Currently, *Streptococcus*, *Porphyromonas*, *Actinomyces*, *Fusarium* and *Fusobacterium* are among the oral microorganisms that have be suggested to be potentially linked to HCC progression (Lu et al., 2016; Li et al., 2020). Regrettably, the mechanism of its role in HCC remains to be further studied. Nevertheless, research has demonstrated a close association between oral microorganisms, specifically *P. gingivalis*, and the development of hepatitis and alcoholic liver disease (Gao et al., 2023). *P. gingivalis* is capable of inducing intestinal microbial dysbiosis and impairing the integrity of the intestinal mucosal barrier, thereby facilitating the migration of enterobacteriaceae to the liver (Nakajima et al., 2015). Additionally, *P. gingivalis* can disrupt the balance between Th17/Treg cells in the intestinal tract, leading to hepatitis and promoting ferroptosis in hepatocytes (Yao et al., 2023). Hence, it is plausible to postulate that the oral microbiota might be implicated in the pathogenesis of liver cancer; however, further empirical evidence is warranted.

### 2.4. Gastric cancer

*H. pylori* infection is a well-established risk factor for gastric cancer, which ranks as the fourth most common cancer globally, with the new incidence of gastric cancer in China was 44% in 2020 (Liu and Dong, 2021). An analysis of the microbiome of gastric cancer in Mexico and China revealed that the dominant bacteria was *H. pylori*, followed by oral microorganisms---*Proteobacteria* and *Firmicutes* (Yu et al., 2017). However, only 1–3% of *H. pylori* infected patients will develop into gastric cancer, so it is reasonable to speculate that other biological factors may be involved in the occurrence and development of gastric cancer (Chen et al., 2019). Study have exhibited elevated abundance of *Peptostreptococcus stomatis*, *Streptococcus anginosus*, *Parvimonas micra*, *Slackia exigua and Dialister pneumosintes* in gastric cancer (Coker et al., 2018). A recent study indicated that more than half of gastric cancer patients tested positive for *Fusobacterium*, *Clostridium* and *Lactobacillus*, suggesting their potential as biomarkers for early diagnosis of gastric cancer (Hsieh et al., 2018). *P. gingivalis* has also been reported to be associated with an increased risk of gastric cancer in Asians (Yang et al., 2022). However, the precise underlying mechanism remains unclear.

### 2.5. Breast cancer

Breast cancer is the fifth most commonly diagnosed tumor worldwide (Sung et al., 2021). Its risk factors primarily include overweight, family genetic history and unhealthy lifestyle such as smoking and excessive alcohol consumption (Liu and Dong, 2021). Previous studies have reported the presence of oral microorganisms in breast milk and speculated on potential transmission routes including: (i) Penetration through the skin and nipple; (ii) Colonization by translocation from the digestive tract and genital tract; (iii) Invasion through blood and lymphatic circulation system, leading to the location in breast lobules and ducts (Urbaniak et al., 2012). However, some scholars suggested that *Proteobacteria* and *Firmicutes* are the predominant microbial groups found in the breast tissue, which exhibit tolerence toward fatty acids infiltration. Therefore, it can be considered that there are unique and diverse microbial groups in the breast (Urbaniak et al., 2014).

For a considerable duration, it has been postulated that alterations in the intestinal microbiota within breast tissue can exert influence on hormone levels and contribute to the pathogenesis of breast cancer (Laborda-Illanes et al., 2020). Meanwhile, limited attention has been given to the oral microbiota in the mammary gland within existing literature. A meta-analysis has revealed a significant association between periodontal disease and oral microbial infection with breast cancer, thereby suggesting that periodontal disease may serves as a potential risk factor for the development of breast cancer (Shao et al., 2018). Another study compared oral rinse samples from 50 breast cancer patients and 20 healthy controls, finding no significant difference in microbial communities (Wang et al., 2017). This result may be constrained by the limited sample size of the included studies. Recently, a sequencing study involving 369 breast cancer cases, 93 non-malignant cases and 419 healthy controls revealed a decrease relative abundance of representative oral microorganisms, namely *P. gingivalis* and *F. nucleatum*, in breast cancer patients (Wu et al., 2022). However, the study did not directly examine the microbiome in breast tumor. Therefore, the association between oral microbiota and breast cancer is somewhat unreliable. Parhi et al. (2020) verified that *F. nucleatum* could spread through blood and colonize breast cancer sites in a FAP2-dependent manner, thereby impairing anti-tumor immunity and promoting the progression of breast cancer, while the corresponding metronidazole antibacterial treatment could delay the progression of tumor. In addition, *F. nucleatum* colonized in breast cancer can also activate NF-κB through TLR4/MyD88 pathway, creating an immunosuppressive TME by recruiting immunosuppressive cells, and promoting the immune escape of breast cancer cells by MYC-dependent up-regulation of PD-L1 and CD47 in breast cancer (Van der Merwe et al., 2021). In conclusion, oral microbiota is involved in the progression of breast cancer, and targeted elimination may be beneficial for the treatment of breast cancer.

### 2.6. Other cancer

Due to the anatomical relationship between oral cavity and digestive tract, there is a higher likelihood of cross-talk between oral microorganisms and tumors in digestive system (Tuominen and Rautava, 2021). In addition to the above-mentioned tumors, oral cancer, esophageal cancer and pancreatic cancer have also been reported to be closely related to oral microbiome (Binder Gallimidi et al., 2015; Fan et al., 2018; Wang et al., 2019; Yamamura et al., 2019). The oral cavity is the main habitat for oral microorganisms, and almost all oral microorganisms have been implicated in the progression of oral cancer.

Oral microorganisms that have been clearly associated with the progression of oral cancer include but are not limited to *Enterococcus faecalis*, *F. nucleatum* and *P. gingivalis* (Zhao et al., 2017; Metsäniitty et al., 2021). *Enterococcus faecalis* can promote the proliferation of oral cancer cells through the H_2_O_2_-mediated EGFR signaling pathway (Boonanantanasarn et al., 2012). *F. nucleatum* promotes the progression of oral cancer by regulating cell cycle, inducing oral inflammation, regulating anti-tumor immune response and promoting epithelial-mesenchymal transition of oral cancer cells (McIlvanna et al., 2021; Shao et al., 2021). The carcinogenic mechanisms of *P. gingivalis* and *F. nucleatum* share similarities (Lafuente Ibáñez de Mendoza et al., 2020; Wen et al., 2020; Yao et al., 2021). Interestingly, an *in vitro* transcriptomic study involving three oral squamous cell carcinoma cell lines, CAL27, SCC4, and SCC25, and three oral commensals, *Streptococcus*, *Neisseria aureus*, and *Haemophilus parainfluis*, with *P. gingivalis* as a positive control, found that only *Streptococcus* exhibited significant antitumor properties alone. The other two commensal bacteria showed the coexistence of pro-tumor and anti-tumor effects, while *P. gingivalis* only had pro-tumor effects on SCC4 cell line (Baraniya et al., 2022). In addition, it has also been reported that *P. gingivalis* can mediate autophagy to induce G1 phase arrest in oral squamous cell carcinoma cells (Cho et al., 2014). Therefore, the exploration of oral microorganisms with anti-tumor effects may provide a new strategy for cancer treatment.

Six studies used 16S rRNA sequencing to analyze saliva and oral swab samples collected from patients with esophageal cancer, revealing significant alterations of oral microorganisms. Although the dominant groups were different, they all suggested that oral microorganisms may be potential screening markers for esophageal cancer (Chen et al., 2015; Wang et al., 2019; Li et al., 2021; Chen X. et al., 2022; Jiang et al., 2022; Nomburg et al., 2022). Currently, *P. gingivalis* and *F. nucleatum* are the primary oral microorganisms reported to be associated with esophageal cancer (Wang et al., 2019; Gao S. et al., 2021; Liu et al., 2021; Lamont et al., 2022). The definite mechanism by which *P. gingivalis* promotes esophageal cancer involves the activation of NF-κB (Meng et al., 2019), PTEN/Akt (Liang et al., 2020), EMT (Chen et al., 2021) and apoptosis resistance signaling pathway (Gao S. et al., 2021), which can regulate proliferation, induce inflammation, create immunosuppressive TME and mediate chemotherapy resistance. *F. nucleatum* can also activate NOD1/RIPK2/NF-κB signaling pathway (Nomoto et al., 2022), AHR/CYP1A1 (Yin et al., 2023), NLRP3 inflammasome (Liang et al., 2022) and autophagy-related signaling pathway (Liu et al., 2021). It also mediates the infiltration of MDSC and regulatory T cells to regulate tumor immunity (Zhang et al., 2021; Liang et al., 2022).

Furthermore, recent studies have highlighted the potential of *P. gingivalis* and *F. nucleatum* as important biomarkers for distinguishing pancreatic cancer patients (Fan et al., 2018; Wei et al., 2020). Notably, the oral microorganism *F. nucleatum* has been found to stimulate the secretion of cytokines, including GM-CSF and CXCL1, thus promoting tumor cell proliferation and migration (Udayasuryan et al., 2022). However, the relationship between other oral microbiota and pancreatic cancer remains elusive.

## 3. Discussion

With the development of technology and interdisciplinary science, the research on microorganisms and cancer has become a hot topic. Due to the anatomical connection, the current reports mainly focus on digestive system tumors, and mainly investigate the common dominant bacteria in the oral microbiome, such as *P. gingivalis* and *F. nucleatum*. This review provides a systematic overview of the association between oral microbiota and the world’s most common malignancies, and summarizes the possible mechanisms reported in the literature. At present, it is believed that oral microorganisms are widely associated with the malignant transformation and progression of tumors. Changes in the abundance of oral microorganisms may be used as potential biomarkers for predicting tumorigenesis, and targeted elimination of related harmful oral microorganisms is expected to become a new strategy for cancer treatment. However, it has also been reported that oral microorganisms play a dual role in tumor biology, which either promotes or inhibits tumor depends on the interaction between microorganisms, host and TME. Oral microorganisms have the ability to colonize extra-oral organs, such as the lungs, colorectum, and stomach (Park et al., 2021). They employ their own virulence factors and metabolites to disrupt the epithelial barrier and extracellular matrix, induce an inflammatory microenvironment and immunosuppressive tumor microenvironment, thereby influencing both local and distant tumors (Li S. et al., 2022; Ma et al., 2023). The anaerobic conditions within the tumor microenvironment further facilitate the accumulation of anaerobic oral microorganisms in tumors (Wu et al., 2021). Intratumoral microorganisms have been found to enhance anti-tumor immunity through activation of the STING signaling pathway, stimulation of T cells and NK cells, formation of intratumoral tertiary lymphoid structures (TLS), as well as microbial-derived antigen presentation mechanisms; Conversely, they can also dampen anti-tumor immune responses by upregulating ROS levels, promoting an anti-inflammatory environment, impairing T cell function, and inducing immunosuppression (Yang et al., 2023). Moreover, spatial transcriptomics analysis revealed a decrease of cytotoxic T cells within regions characterized by higher bacterial accumulation in tumors, accompanied by more potent immunosuppressive effects (Galeano Niño et al., 2022). Studies on oral microbiota also present a dual perspective: The Fap2 protein, expressed by *F. nucleatum*, interacts with human inhibitory receptors TIGIT to facilitate the evasion of tumor cells from NK cells and T cells (Gur et al., 2015). Additionally, it has been demonstrated that *F. nucleatum* can activate the STING signaling pathway and enhance the expression of PD-L1, thereby exhibiting heightened susceptibility toward PD-L1 immune blockade therapy (Gao Y. et al., 2021). Therefore, the conflicting conclusions need to be further verified, and the beneficial microorganisms against cancer need to be further explored to broaden the treatment options for cancer.

## Author contributions

ZL, W-JL, Y-YZ, and G-TY: conceptualization. ZL and W-JL: formal analysis and writing the original draft. ZL, WJ-L, HCu, K-LZ, HCh, Y-YZ, and G-TY: writing review and editing. Y-YZ and G-TY: supervision and funding acquisition. All authors contributed to the article and approved the submitted version.
